# Elevated Liver Enzymes With Positive Hepatitis B Serology After Intravenous Immunoglobulin Treatment of Guillain-Barré Syndrome

**DOI:** 10.14309/crj.0000000000001207

**Published:** 2023-12-09

**Authors:** Shivam Patel, Kim Minh Ngoc Le, Sujith Puskoor, Judith Amaning

**Affiliations:** 1 Texas A&M University School of Medicine, Bryan, TX; 2 Department of Gastroenterology, Baylor Scott & White Medical Center Round Rock, Round Rock, TX; 3 Department of Internal Medicine, Baylor Scott & White Medical Center Round Rock, Round Rock, TX

**Keywords:** IVIG, Guillain-Barré syndrome, hepatitis B serology, liver enzyme elevation

## Abstract

Intravenous immunoglobulin (IVIG) is used to treat multiple conditions, one of which is Guillain-Barré syndrome. Despite its multiple benefits, IVIG can cause a wide variety of side effects, most of which resolve with supportive care. We present a case in which a patient with new-onset Guillain-Barré syndrome was treated with IVIG and subsequently developed an acute elevation in liver enzymes with positive hepatitis B serology.

## INTRODUCTION

Intravenous immunoglobulin (IVIG) is the standard of care for Guillain-Barré syndrome (GBS), if patients are within 4 weeks of symptom onset. Adverse effects are associated with renal, vascular, cardiac, dermatological, and neurological systems.^1–3^ However, gastrointestinal adverse effects, such as drug-induced liver injury and transfer of hepatitis antibodies, are less commonly observed.

## CASE REPORT

A 49-year-old woman with a medical history of resolved hepatitis A, gastric ulcers, and a cholecystectomy presented to the emergency department with a 2-day history of ascending sensation changes in her bilateral lower extremities extending to her lower back and saddle area. In addition, she experienced weakness and new-onset urinary incontinence. She denied any trauma, preceding upper respiratory infection, or gastrointestinal illness.

She was evaluated in the emergency department the day before admission for the abovementioned symptoms before she developed urinary incontinence. She was given 1 dose each of oral diazepam 5 mg, intravenous fentanyl 5 mg, and intravenous ketorolac 15 mg. The patient was discharged with etodolac 400 mg twice daily, which she states she never took. Her home supplements were collagen and hyaluronic acid supplements.

After physical examination, baseline laboratory tests, lumbar puncture, and magnetic resonance imaging of the lumbar spine showing enhancement in the conus medullaris, she was diagnosed with GBS. She was admitted and started on 2 g/kg of IVIG (divided over 4 days), gabapentin, and as-needed 650 mg acetaminophen.

On admission, complete blood count and comprehensive metabolic panel were unremarkable. There were no previous acute hepatitis laboratory tests in her medical record. After her initial dose of IVIG, her neurological symptoms started to improve. Repeat laboratory tests the morning after initiating IVIG treatment showed elevated liver function tests (LFTs). The increase in LFTs also corresponded to an increase in total protein due to IVIG (Table 1).

**Table 1.** Patient's trends in LFTs and total protein on admission, after days 1–4 of IVIG treatment, before discharge, and 2 months after

	Admission	Day 1	Day 2	Day 3	Day 4	Discharge	2 mo later
AST (U/L)	27	166	244	669	758	614	41
ALT (U/L)	39	134	233	503	690	653	58
ALP (U/L)	84	119	151	222	324	322	124
Total protein (g/dL)	7.9	7.3	8.4	8.8	8.7	8.2	7.8

ALP, alkaline phosphatase; ALT, alanine aminotransferase; AST, aspartate aminotransferase; IVIG, intravenous immunoglobulin; LFT, liver function test.

A hepatitis C antibody, hepatitis C RNA viral load, coagulation studies, antimitochondrial antibody levels, liver kidney microsomal antibody levels, ceruloplasmin levels, alpha-1 antitrypsin levels, and celiac serology were unremarkable. Epstein-Barr viral panel, varicella panel, herpes simplex viral panel, and cytomegalovirus panel were unremarkable for acute infection. A right upper quadrant ultrasound with Doppler revealed patent hepatic and portal vasculature. Ferritin was elevated likely because of the acute underlying condition of GBS; transferrin saturation indicated iron deficiency anemia. Anti-smooth muscle antibody was slightly positive at 1:40. The total hepatitis B core antibody returned positive, as well as total surface antibodies.

Hepatitis Be antigen and antibody, hepatitis B DNA viral load, hepatitis B core immunoglobulin M antibody, and hepatitis B surface antigen were all unremarkable. The only other new medication during the admission was gabapentin, and the patient received minimal amounts of acetaminophen. Subsequent laboratory tests in the following days showed an upward trend in LFTs. Despite this upward trend, the decision was made to complete the recommended 4 days of IVIG treatment of GBS because the patient's symptoms were improving with the IVIG infusions and hepatic function remained intact. Follow-up of total anti-HBc showed a borderline result, indicating a downward trend when compared with the previous laboratory test results. The rest of the patient's LFTs also trended downward after she completed the IVIG treatment and before discharge. Repeat laboratory tests 2 months later demonstrated near normalization of all liver enzymes; total protein and ferritin levels completely normalized. Further laboratory test results are not available because she subsequently followed up with an outside clinic, but she reported 5 months later that all her laboratory test results have normalized.

## DISCUSSION

Elevated liver enzymes after IVIG treatment of GBS is not extensively reported in the current literature. There is 1 case report that mentioned an increase in liver enzymes after IVIG treatment of GBS.^4^ The report describes several patients who experienced elevated LFTs; however, the extent of the elevation was not as substantial as our patient's. In addition, the formulation used contained maltose as a stabilizing agent, which the study concluded was the likely cause of the elevated LFTs. Gammagard liquid, which was the IVIG formulation used in our patient, contains glycine and is not known to cause elevated liver enzymes.^4,5^ In our case, it was important to rule out other causes of liver injury.

Our patient was administered 3 drugs during her initial emergency department visit. Aminotransferase elevations greater than 3-fold with ketorolac occurs in <1% of patients, and it is currently unproven whether ketorolac causes a clinically apparent liver injury.^6^ Oral diazepam is rarely associated with elevated liver enzymes, and the onset of injury is between 1 and 6 months.^7^ Fentanyl has not been linked to cases of liver injury.^8^ Our patient confirmed that she did not start taking etodolac. Her liver enzyme elevation occurred before she received acetaminophen. She was started on gabapentin; however, this usually causes a cholestatic pattern of injury, and the latency period is 1–8 weeks.^9^

Our patient also had elevated hepatitis B core antibodies. All other hepatitis B laboratory tests indicated previous immunity and no evidence of reactivation or active infection. The positive anti-HBc was most likely transferred passively from IVIG. There are case reports that mention patients receiving IVIG who had positive hepatitis B antibodies, which was the result of passive transfer; liver enzyme elevation was observed in some of the patients.^3,10–14^

We initially suspected that our patient's elevated liver enzymes were due to drug-induced liver injury, and her positive anti-HBc antibodies were transferred to her bloodstream from the IVIG. Using the Roussel Uclaf Causality Assessment Model, her score of 5 indicated IVIG possibly caused a liver injury. Alternative causes of elevated liver enzymes include a hepatic reaction to the transfer of viral antibodies or an autoimmune hepatic drug reaction to IVIG, the latter due to the positive anti-smooth muscle antibody. Before starting IVIG, a baseline acute hepatitis panel may be helpful in evaluating the significance of positive antibody titers, especially in patients without a confirmed prior infection or vaccination history.^10,13,14^ Although the extent of liver enzyme elevation can be significant, as seen in our patient, it usually does not require aggressive treatment, and watchful waiting with supportive care is likely sufficient.

## DISCLOSURES

Author contributions: S. Patel, KMN Le, and S. Puskoor all contributed to writing and editing the manuscript. J. Amaning is the Gastroenterology attending physician who reviewed and edited the case report. S. Puskoor, KMN Le, and J. Amaning were involved in the direct care of the patient. KMN Le is the article guarantor.

Financial disclosure: None to report.

Informed consent was obtained for this case report.
